# Supplementary material for: A recent increase in global wave power as a consequence of oceanic warming
Source: Nat Commun. 2019 Jan 14;10:205. doi: 10.1038/s41467-018-08066-0 (PMC6331560; doi:10.1038/s41467-018-08066-0)
Supplement: Supplementary file 1 — Supplementary Information [file 41467_2018_8066_MOESM1_ESM.pdf]

# A recent increase in global wave power as a consequence of oceanic warming


## Reguero et al.


## Supplementary Information


Correspondence to:

borjagreguero@gmail.com

or losadai@unican.es

# Content

Supplementary Figure 1. Comparison of numerical data with satellite-derived data. .................... 3

Supplementary Figure 2. Spatial mean annual Wave Power calculated by latitudinal bands. ....... 4

Supplementary Note 1. Extreme wave heights. ............................................................................ 5

Supplementary Figure 3. 100-year significant wave height. ......................................................... 6

Supplementary Figure 4. Lagged correlations. .............................................................................. 7

Supplementary Figure 5. Spatial changes in Mean Wave Power. ................................................. 8

Supplementary Note 2. El Niño events in the decadal patterns. .................................................... 9

Supplementary Figure 6. Changes in mean Wave Power for different decadal periods. ............. 10

Supplementary Figure 7. Changes in Sea Surface Temperature anomalies for different decadal periods. ......................................................................................................................................... 11

Supplementary Table 1. Inter-regional correlations of Sea Surface Temperature. ....................... 12

Supplementary Table 2. Contemporaneous inter-regional correlations. ..................................... 13

Supplementary Table 3. Contemporaneous inter-regional correlations in the satellite era. ......... 14

Supplementary Table 4. Inter-regional correlations in the satellite era. ....................................... 15

Supplementary Table 5. Inter-regional correlations of non-autocorrelation residuals. ................ 16

Supplementary Table 6. Inter-regional correlations of non-autocorrelation residuals during the satellite era, 1979-2008. ............................................................................................................ 17

Supplementary Discussion ........................................................................................................... 18

Supplementary Code. Mann-Kendall test following the method in Wang and Swail (2001) ...... 21

Supplementary References ........................................................................................................... 23

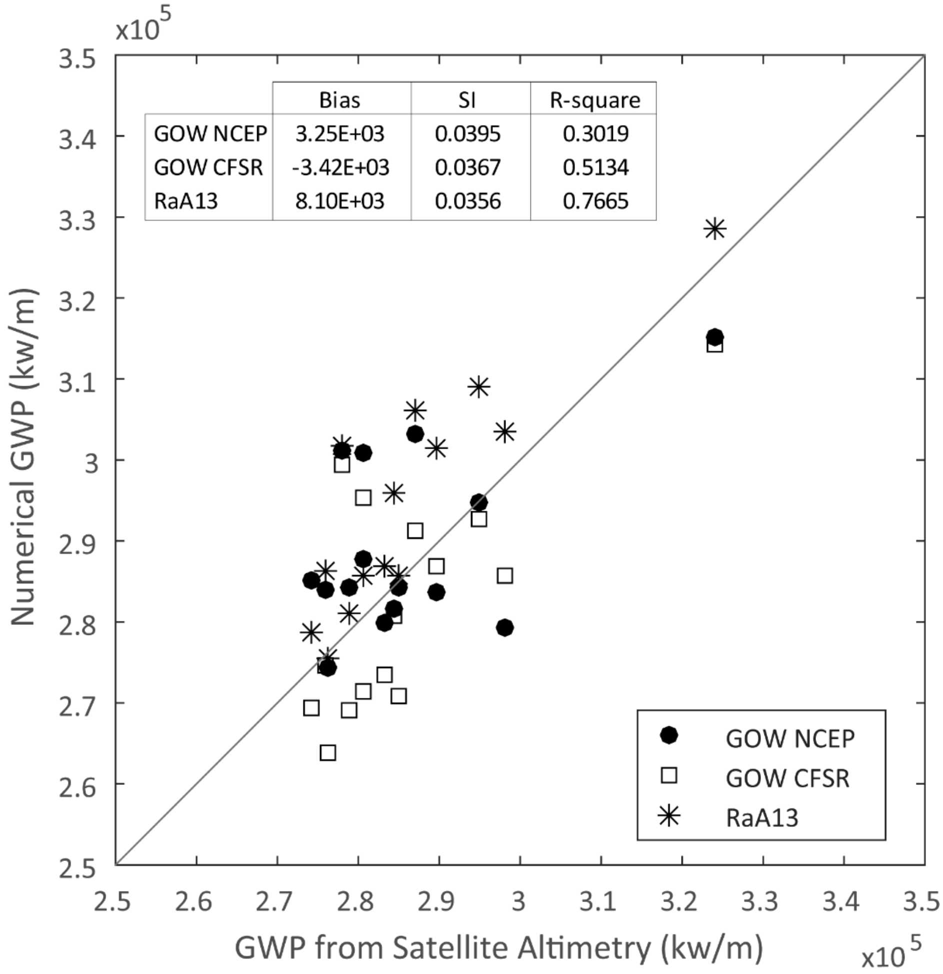


**Supplementary Figure 1. Comparison of numerical data with satellite-derived data.** Comparison of the Global Wave Power (GWP) calculated from satellite altimetry (x-axis) and the numerically derived Global Wave Power (y-axis) for the wave datasets used in the analysis. The diagnostic statistics for comparing hindcast performance (y) with respect to the satellite altimetry Global Wave Power (x) are calculated as follows: $Bias = \bar{x} - \bar{y}$ ; Residual Scatter Index: $SI = \frac{RMSE}{\bar{x}}$, where $RMSE = \sqrt{\frac{1}{n_d}\sum_{i=1}^{n_d}(x_i - y_i)^2}$; and R-square as the Coefficient of Determination of the linear regression between x and y.

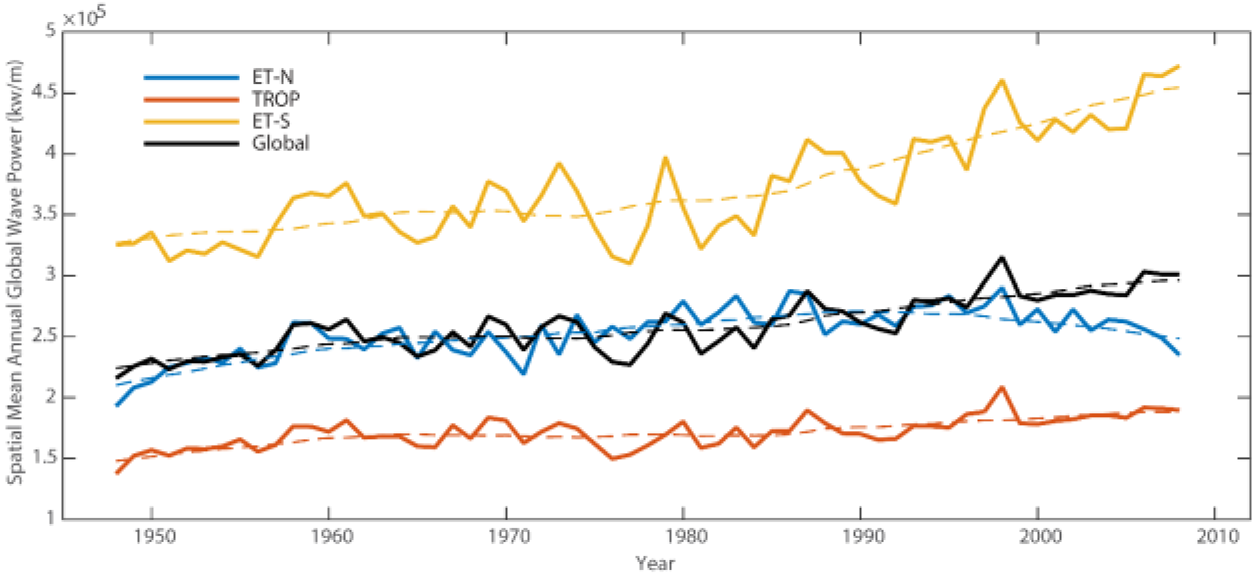


**Supplementary Figure 2. Spatial mean annual Wave Power calculated by latitudinal bands.** The dashed lines indicate the 10-year moving averages. The Wave Power in each region is calculated as the spatial average of each historical Wave Power time series between the 30° north and south latitudinal limits.

**Supplementary Note 1. Extreme wave heights.**

The spatial pattern for the 1-in-100-year significant wave heights is consistent with previous works [1–3]. In particular, a similar extreme analysis was performed by Izaguirre et al. using the same extreme value model, but the altimeter observations revealed patterns for the significant wave height for a return period of 100 years in close agreement with those obtained here [3]. This indicates that the statistical behavior of the wave reanalysis is well modeled for extreme wave heights compared to satellite altimetry observations.

However, Supplementary Figure 2 also demonstrates that the WP is a wave parameter that represents different properties of the wave climate in each oceanic basin. Figure 1 in the main text shows that the Atlantic is not one of the most energetic basins, although it has some of the most extreme wave heights (Supplementary Figures 2 and 3).

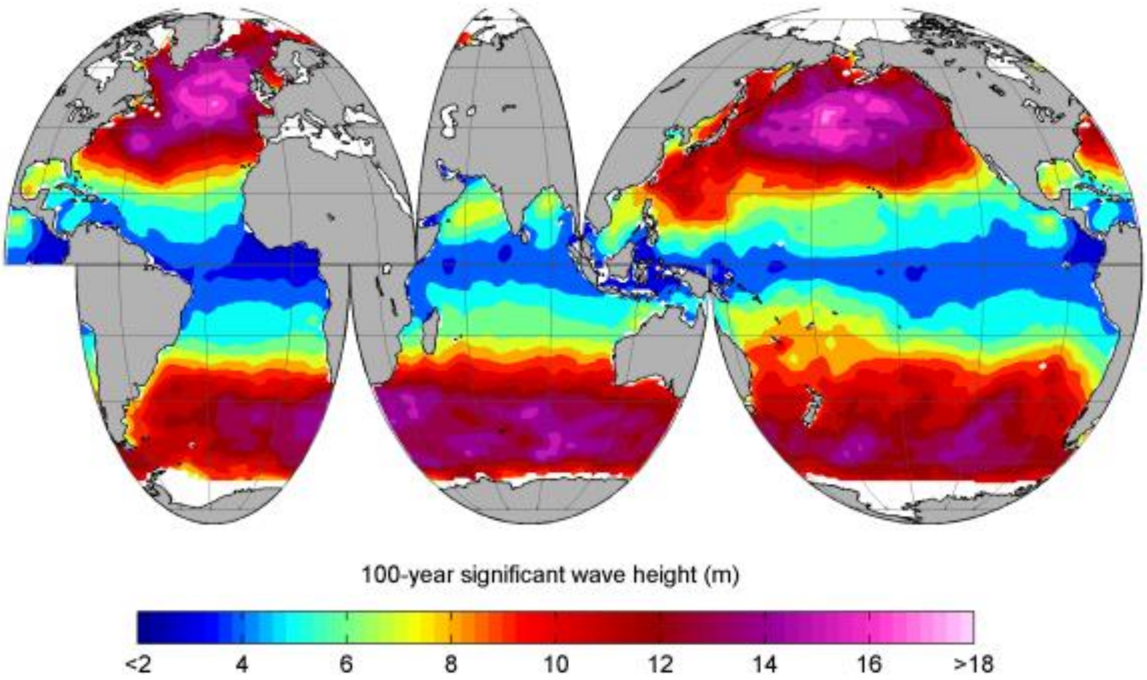


**Supplementary Figure 3. 100-year significant wave height.** Significant wave height for a return period of 100 years, calculated via the annual maxima method with the 61-year GOW wave dataset using a generalized extreme value distribution [4].

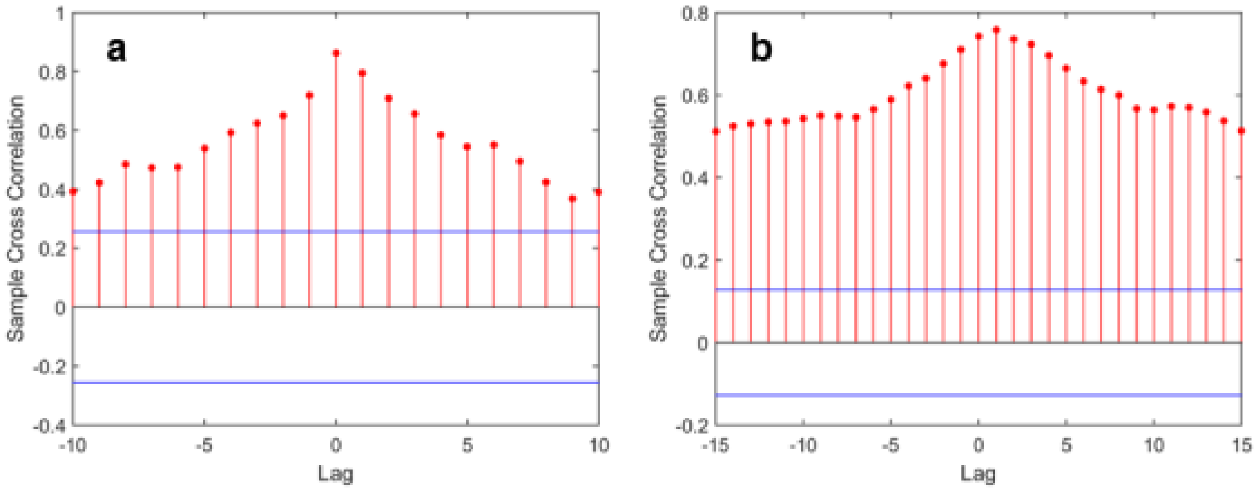


**Supplementary Figure 4. Lagged correlations.** Lagged-correlation analysis of Global Wave Power and Sea Surface Temperature anomalies for (**a**) annual and (**b**) seasonal time series. Time lags are expressed in years (**a**) and seasons (**b**).

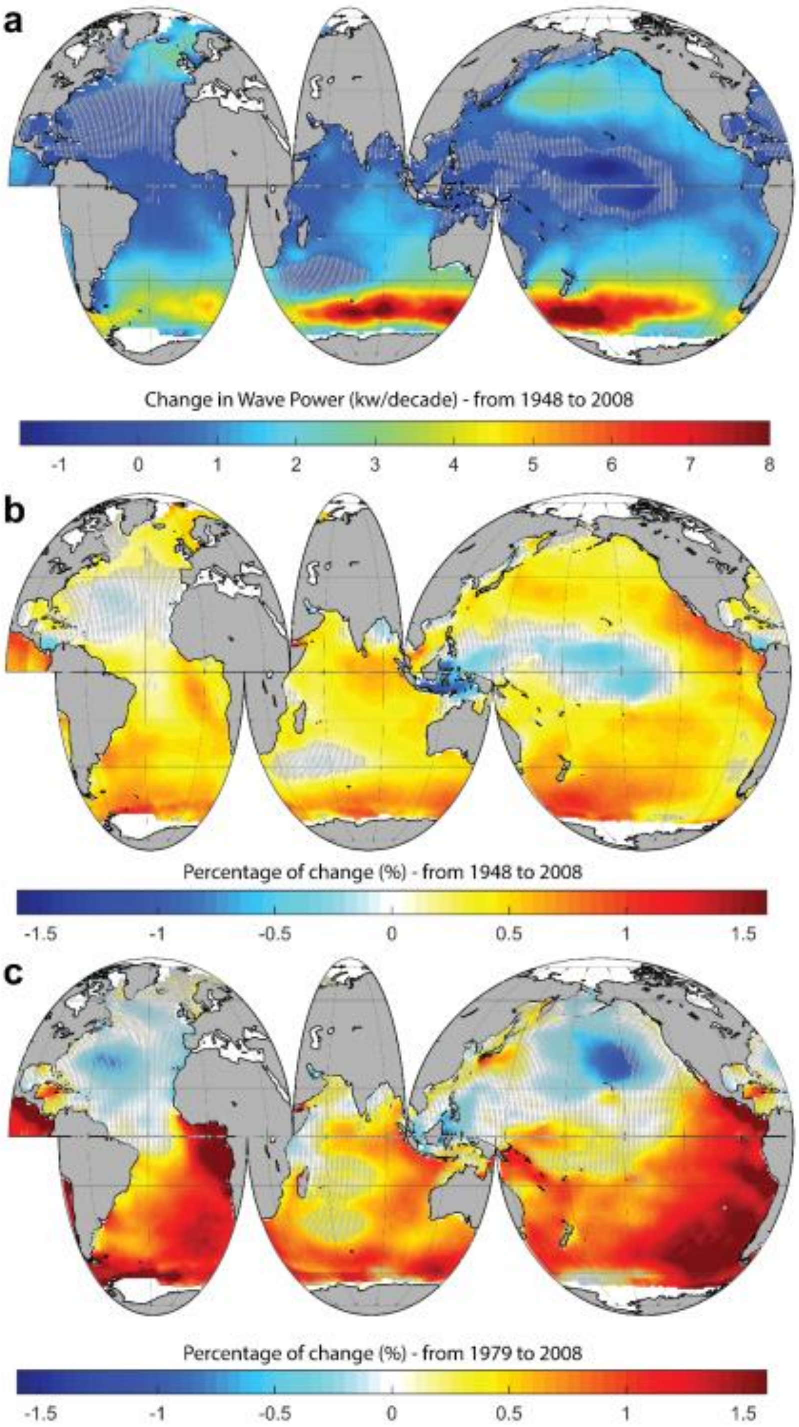


**Supplementary Figure 5. Spatial changes in Mean Wave Power.** Mean change in Wave Power over the historical time span (1948-2008) expressed in absolute values per decade (a), percentage per year (b), and percentage per year but only for the time span covering satellite-derived wave measurements (after 1985). The hatched areas mark regions that are not significant at the 95% level.

**Supplementary Note 2. El Niño events in the decadal patterns.**

Both the 1983 and 1998 El Niño events are shown in Supplementary Figures 6-**c** and **d** (blue tones in the central Pacific), but the 1998 El Niño is more intense, as it is clearly marked by a strong difference in standard deviation (Supplementary Figure 6-**f**). The decadal changes in WP depicted in Supplementary Figure 7-**d** are consistent with the identified patterns based on the climate indices in Figure 7 in the main manuscript for Niño3 and the AMO, which was dominant in the decade 1989-1998 (Supplementary Figure 6-**d**).

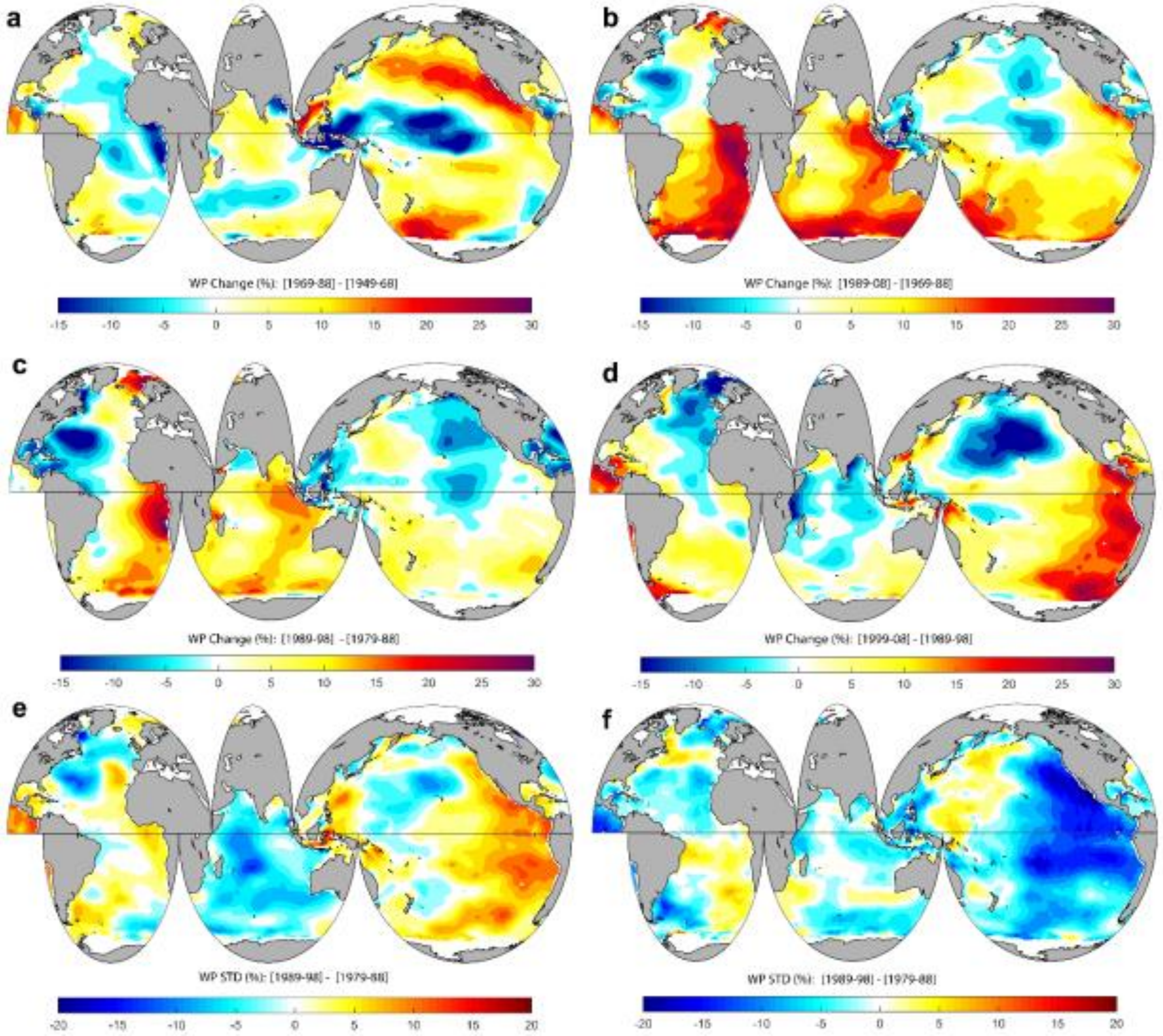


**Supplementary Figure 6. Changes in mean Wave Power for different decadal periods.** Change in mean Wave Power (WP) by periods of 20 years from 1949-1968 to 1969-1988 (**a**) and from 1969-1988 to 1989-2008 (**b**). Changes in mean Wave Power by periods of 10 years from 1979-1988 to 1989-1998 (**c**) and from 1989-1998 to 1999-2008. Standard deviations (STDs) from 1979-1988 to 1989-1998 (**e**) and from 1989-1998 to 1999-2008 (**f**). Changes are expressed as the percentage of change with respect to the mean value for the entire time series (1949-2008 for **a** and **b** and 1979-2008 for **c** and **d**), for example, $(X_2 - X_1)/Z$, where $X_i$ corresponds to the mean WP for timespan *i* (10 or 20 years), and Z represents the mean value for the whole period.

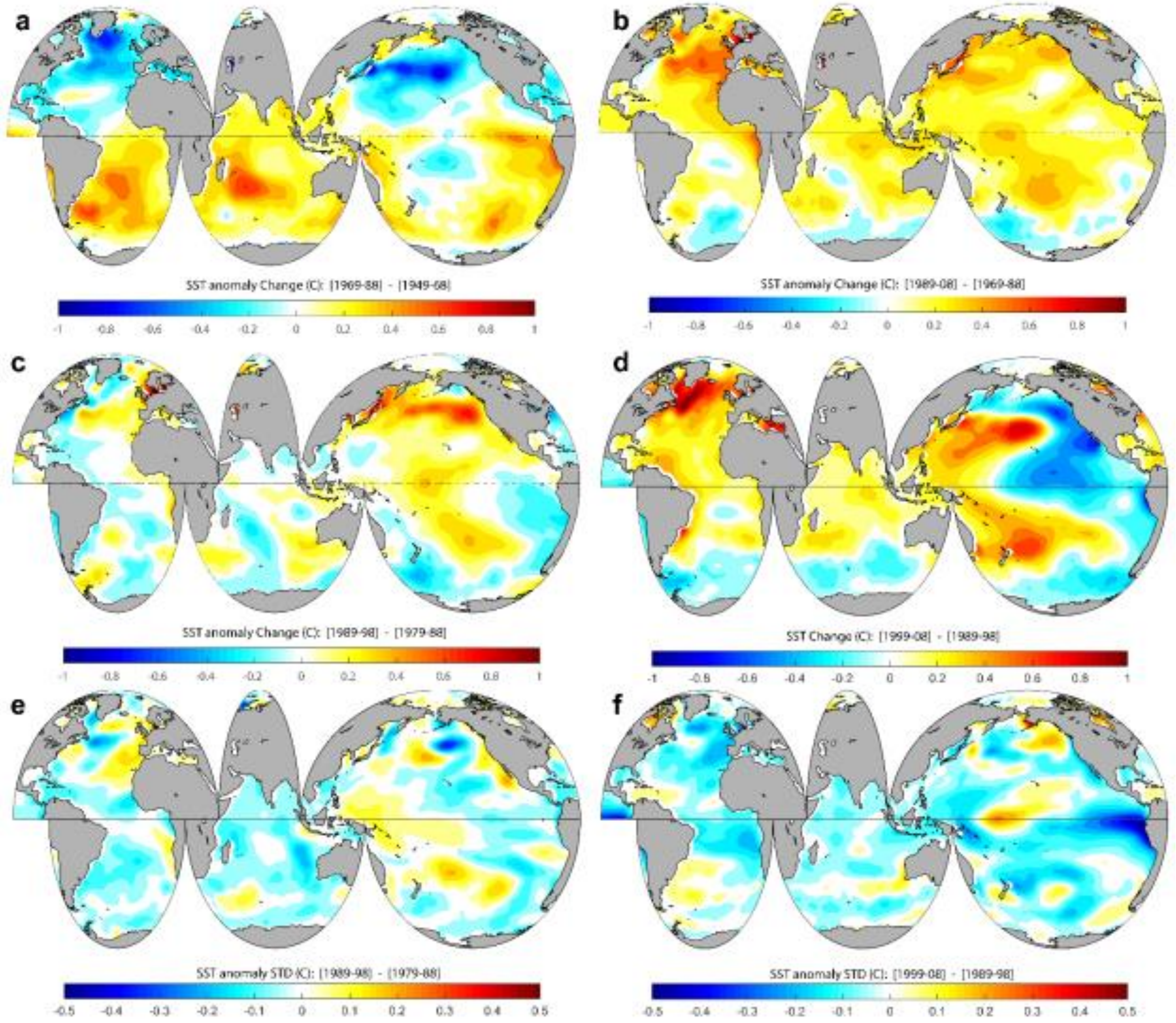


**Supplementary Figure 7. Changes in Sea Surface Temperature anomalies for different decadal periods.** Change in mean Sea Surface Temperature (SST) anomaly by periods of 20 years from 1949-1968 to 1969-1988 (**a**) and from 1969-1988 to 1989-2008 (**b**). Changes in mean Sea Surface Temperature anomaly by periods of 10 years and changes in the satellite era from 1979-1988 to 1989-1998 (**c**) and from 1989-1998 to 1999-2008. Standard deviations (STDs) from 1979-1988 to 1989-1998 (**e**) and from 1989-1998 to 1999-2008 (**f**). Changes are expressed in degrees above mean values. The differences are the mean anomaly during one time interval from that of the previous one, for example, $(X_2 - X_1)$, where $X_i$ corresponds to the mean SST anomaly over time span *i* (10 or 20 years).

| | ETNP | ETNP | ETNP | ETNP | ETNP | ETNP | ETNP | ETNP |
|---|---|---|---|---|---|---|---|---|
| ETNP | 1.00 | 0.21 | 0.20 | 0.48 | 0.26 | - | 0.17 | 0.15 |
| TPAC | | 1.00 | 0.51 | 0.29 | 0.71 | 0.46 | 0.79 | 0.50 |
| ETSP | | | 1.00 | 0.24 | 0.56 | 0.69 | 0.66 | 0.68 |
| ETNA | | | | 1.00 | 0.48 | - | 0.29 | 0.24 |
| TATL | | | | | 1.00 | 0.50 | 0.80 | 0.51 |
| ETSA | | | | | | 1.00 | 0.60 | 0.71 |
| TIOC | | | | | | | 1.00 | 0.67 |
| ETSI | | | | | | | | 1.00 |

**Supplementary Table 1. Inter-regional correlations of Sea Surface Temperature.** Correlation of the non-autocorrelated residuals of Sea Surface Temperatures (SST) across ocean sub-basins. High correlations (above 0.5) are highlighted in bold italics. Regions: extratropical North Pacific (ETNP), tropical Pacific (TPAC), extratropical South Pacific (ETSP), extratropical North Atlantic (ETNA), tropical Atlantic (TATL), extratropical South Atlantic (ETSA), tropical Indian Ocean (TIOC) and extratropical south Indian Ocean (ETSI).

| 1948-2008 | | WP | | | | | | | |
|---|---|---|---|---|---|---|---|---|---|
| | | ETNP | TPAC | ETSP | ETNA | TATL | ETSA | TIOC | ETSI |
| SST | ETNP | - | 0.16 | 0.25 | - | 0.19 | 0.29 | 0.18 | 0.21 |
| | TPAC | 0.48 | 0.48 | 0.39 | 0.20 | 0.32 | 0.52 | 0.39 | 0.52 |
| | ETSP | 0.37 | 0.37 | 0.49 | - | 0.26 | 0.44 | 0.42 | 0.39 |
| | ETNA | - | 0.22 | 0.38 | - | 0.28 | 0.46 | 0.23 | 0.39 |
| | TATL | 0.33 | 0.48 | 0.62 | 0.17 | 0.39 | 0.60 | 0.43 | 0.55 |
| | ETSA | 0.39 | 0.28 | 0.47 | 0.16 | 0.17 | 0.38 | 0.38 | 0.37 |
| | TIOC | 0.43 | 0.51 | 0.58 | 0.21 | 0.37 | 0.58 | 0.46 | 0.55 |
| | ETSI | 0.38 | 0.29 | 0.45 | 0.15 | 0.23 | 0.45 | 0.34 | 0.45 |

**Supplementary Table 2. Contemporaneous inter-regional correlations.** Correlation between the Sea Surface Temperature (SST) and regional Wave Power (WP) during 1948-2008. Seasonal correlations between average Sea Surface Temperature anomalies in a given sub-basin (rows) and Wave Power in a given sub-basin (columns). The values correspond to Pearson's linear correlation coefficient at the 95% confidence interval. The ocean sub-basins correspond to: extratropical north Pacific (ETNP), tropical Pacific (TPAC), extratropical south Pacific (ETSP), extratropical north Atlantic (ETNA), tropical Atlantic (TATL), extratropical south Atlantic (ETSA), tropical Indian Ocean (TIOC) and extratropical south Indian Ocean (ETSI).

| 1979-2008 | | WP | | | | | | | |
|---|---|---|---|---|---|---|---|---|---|
| | | ETNP | TPAC | ETSP | ETNA | TATL | ETSA | TIOC | ETSI |
| SST | ETNP | | | 0.288 | | 0.222 | 0.364 | | 0.247 |
| | TPAC | 0.238 | 0.532 | 0.259 | | | 0.263 | | 0.339 |
| | ETSP | | - | | | | 0.190 | | 0.217 |
| | ETNA | | 0.365 | 0.610 | | 0.300 | 0.543 | 0.248 | 0.399 |
| | TATL | | 0.501 | 0.625 | | 0.234 | 0.468 | 0.193 | 0.369 |
| | ETSA | -0.193 | | | | | | 0.189 | |
| | TIOC | | 0.542 | 0.478 | | | 0.325 | | 0.326 |
| | ETSI | | 0.213 | 0.233 | | 0.221 | 0.323 | 0.202 | 0.350 |

**Supplementary Table 3. Contemporaneous inter-regional correlations in the satellite era.** Correlation between the Sea Surface Temperature (SST) and regional Wave Power (WP) during the satellite era, from 1979-2008. Seasonal correlations between average Sea Surface Temperature anomalies in a given sub-basin (rows) and Wave Power in a given sub-basin (columns). The values correspond to Pearson's linear correlation coefficient at the 95% confidence interval. The ocean sub-basins correspond to: extratropical north Pacific (ETNP), tropical Pacific (TPAC), extratropical south Pacific (ETSP), extratropical north Atlantic (ETNA), tropical Atlantic (TATL), extratropical south Atlantic (ETSA), tropical Indian Ocean (TIOC) and extratropical south Indian Ocean (ETSI).

| 1979-2008 | | WP | | | | | | | |
|---|---|---|---|---|---|---|---|---|---|
| | | ETNP | TPAC | ETSP | ETNA | TATL | ETSA | TIOC | ETSI |
| SST | ETNP | -0.28 (-2) | 0.19 (3) | 0.41 (-2) | | 0.35 (-1) | 0.46 (-1) | 0.23 (-2) | 0.36 (3) |
| | TPAC | 0.24 (0) | 0.53 (0) | 0.42 (3) | -0.19 (3) | | 0.32 (3) | 0.23 (2) | 0.37 (-1) |
| | ETSP | | 0.25 (-2) | 0.34 (3) | | | 0.23 (1) | 0.29 (-3) | 0.27 (3) |
| | ETNA | -0.23 (1) | 0.47 (-1) | 0.66 (-1) | -0.19 (-1) | 0.33 (3) | 0.54 (0) | 0.28 (-1) | 0.45 (-1) |
| | TATL | -0.28 (3) | 0.51 (-1) | 0.63 (1) | | 0.35 (1) | 0.47 (0) | 0.22 (1) | 0.46 (-2) |
| | ETSA | -0.19 (0) | | 0.24 (-1) | | 0.19 (-2) | 0.22 (2) | 0.19 (0) | |
| | TIOC | -0.19 (3) | 0.60 (-1) | 0.53 (1) | -0.27 (3) | | 0.35 (-1) | 0.21 (2) | 0.38 (-2) |
| | ETSI | -0.20 (-3) | 0.22 (-1) | 0.23 (0) | | 0.29 (-3) | 0.35 (-3) | 0.32 (3) | 0.35 (0) |

**Supplementary Table 4. Inter-regional correlations in the satellite era.** Lagged-correlation analysis of regional time series during the satellite era, 1979-2008. Seasonal correlations between average Sea Surface Temperature (SST) anomalies in a given sub-basin (rows) and Wave Power (WP) in a given sub-basin (columns). The first value corresponds to Pearson's linear correlation coefficient at the 95% confidence interval, while the second value (in parentheses) gives the time lag for the maximum correlation found. The values in parentheses represent the time lag in terms of the number of seasons. Equivalent numbers for the satellite era can be found in the Supplementary Information. The ocean sub-basins correspond to: extratropical north Pacific (ETNP), tropical Pacific (TPAC), extratropical south Pacific (ETSP), extratropical north Atlantic (ETNA), tropical Atlantic (TATL), extratropical south Atlantic (ETSA), tropical Indian Ocean (TIOC) and extratropical south Indian Ocean (ETSI).

| 1948-2008 | | Non-autocorrelated residual WP | | | | | | | |
|---|---|---|---|---|---|---|---|---|---|
| | | ETNP | TPAC | ETSP | ETNA | TATL | ETSA | TIOC | ETSI |
| Non-autocorrelated residual SST | ETNP | -0.21 (-1) | | | | 0.18 (-1) | | -0.15 (-3) | |
| | TPAC | 0.13 (0) | 0.16 (0) | -0.18 (0) | | | 0.18 (3) | | |
| | ETSP | | | | | | | 0.15 (-3) | |
| | ETNA | | 0.16 (-1) | | 0.18 (-3) | | 0.14 (0) | 0.19 (-1) | 0.17 (-1) |
| | TATL | 0.20 (2) | 0.14 (2) | | -0.14 (2) | 0.15 (1) | | | |
| | ETSA | | | -0.16 (2) | -0.18 (-3) | -0.19 (0) | | | |
| | TIOC | | 0.17 (-1) | 0.14 (3) | 0.14 (-1) | -0.13 (-2) | 0.16 (0) | -0.14 (-1) | |
| | ETSI | | -0.14 (-3) | | | | | 0.15 (3) | |

**Supplementary Table 5. Inter-regional correlations of non-autocorrelation residuals.** Seasonal correlations between non-autocorrelation residuals of Sea Surface Temperature (SST) in a given sub-basin (rows) and non-autocorrelation residuals of Wave Power (WP) in a given sub-basin (columns). The values in brackets indicate time lags in terms of the number of seasons. No lagged values beyond three seasons are considered to be representative. Regions: extratropical North Pacific (ETNP), tropical Pacific (TPAC), extratropical South Pacific (ETSP), extratropical North Atlantic (ETNA), tropical Atlantic (TATL), extratropical South Atlantic (ETSA), tropical Indian Ocean (TIOC) and extratropical south Indian Ocean (ETSI).

| 1979-2008 | | Non-autocorrelated residual WP | | | | | | | |
|---|---|---|---|---|---|---|---|---|---|
| | | ETNP | TPAC | ETSP | ETNA | TATL | ETSA | TIOC | ETSI |
| Non-autocorrelated residual SST | ETNP | -0.22 (-2) | -0.21 (-2) | | | 0.25 (-1) | | | |
| | TPAC | | -0.25 (-3) | -0.23 (-3) | | 0.18 (-3) | 0.22 (3) | | 0.21 (0) |
| | ETSP | 0.24 (-2) | | | | | | 0.20 (-1) | |
| | ETNA | | 0.25 (-1) | 0.27 (-1) | 0.19 (-3) | | | | |
| | TATL | 0.28 (-2) | 0.19 (-2) | 0.19 (-1) | | | | | -0.19 (2) |
| | ETSA | | | -0.31 (2) | | 0.22 (-2) | | -0.20 (-3) | |
| | TIOC | 0.21 (-2) | 0.20 (1) | 0.22 (1) | | -0.23 (-2) | | 0.19 (3) | |
| | ETSI | -0.22 (-3) | | | 0.18 (-2) | 0.19 (-3) | | 0.20 (3) | 0.25 (0) |

**Supplementary Table 6. Inter-regional correlations of non-autocorrelation residuals during the satellite era, 1979-2008.** Seasonal correlations between non-autocorrelation residuals of Sea Surface Temperature (SST) in a given sub-basin (rows) and non-autocorrelation residuals of Wave Power (WP) in a given sub-basin (columns). The first value corresponds to Pearson's linear correlation coefficient at the 95% confidence interval, while the second value (in parentheses) gives the time lag for the maximum correlation found. The values in parentheses represent the time lag in terms of the number of seasons. Equivalent numbers for the satellite era can be found in the Supplementary Information. The ocean sub-basins correspond to: extratropical north Pacific (ETNP), tropical Pacific (TPAC), extratropical south Pacific (ETSP), extratropical north Atlantic (ETNA), tropical Atlantic (TATL), extratropical south Atlantic (ETSA), tropical Indian Ocean (TIOC) and extratropical south Indian Ocean (ETSI).

## Supplementary Discussion

Semi-empirical relationships have been used before to explain the responses in variables related to global warming, for example, sea level rise [5]. In this study, the projections of GWP based on direct regression with SST are in the order of magnitude of dynamic projections of wave climate, but a direct regression with SST needs to be taken with caution. There are important factors that prevent the direct extrapolation between SST and GWP at the end of the century. First, there are underlying uncertainties in the climatic mechanisms and dynamic effects of climate change that are not reflected in the historical time span. These uncertainties include the effects on wind, and thus WP, due to the weakening of the Atlantic Meridional Overturning Circulation (11% in RCP2.6 and 34% in RCP8.5, on average) and the changes in Arctic sea-ice cover, which can modify where waves are generated and how they reach coastal zones. Furthermore, we have also shown in Figures 7 and 8 that variations in interannual climate patterns are a key factor in WP variability, and these patterns could change in the future in terms of intensity and frequency [6].

The inter-regional correlations by ocean basins and the response of WP with SST-based climate indices (Figure 7) indicate that SSTs in the different basins affect WP across the different oceans. The SST-WP inter-regional correlation patterns found in Figure 8 align with teleconnections between regional SST changes and storm activity [7,8]. The strongest SST-WP correlation between the tropical Atlantic SST and WP in the extratropical South Pacific and South Atlantic are explained by ocean-atmospheric connections that relate the seasonal fields of sea level pressure, wind and SST temperature in the tropical Atlantic, eastern Pacific and Indian oceans [9]. Atlantic SSTs influence remote tropical storm activity in the eastern-central Pacific through a Walker circulation-type response analogous to the ENSO-Atlantic teleconnection [10]. Atlantic SSTs are also connected to the NAO and the AMO (Figure 7-**c**). First, North Atlantic SSTs drives a NAO-like response [11]: a SST warming/cooling in the subpolar and the eastern tropical North Atlantic leads a negative/positive phase of the NAO, which is the most influential pattern on the North Atlantic wave climate [12]. Therefore, a North Atlantic warming represents a less energetic local wave climate, consistently with the lack of correlations found in Figure 8 for SST and WP in the mid and extratropical North Atlantic. However, a negative NAO is preceded by an AMO-like SST anomaly, which influences WP in the Southern Ocean through ocean-atmosphere

teleconnections. This is shown in Figure 8 through correlations between the North Atlantic SSTs and the Southern Ocean WP in the satellite era. Second, the AMO is suggested to be primarily forced by Atlantic SSTs in the northern subtropics [11] and enhances WP across the Southern Ocean as shown based on correlations for the AMO index in Figure 7-**d** and in the decadal changes from 1989 to 2008. Particularly during the last decade analyzed (1999-2008), the warming of the Atlantic (i.e. positive phase of the AMO) decreased the WP in the North Atlantic and North Pacific but strongly enhanced the WP in the rest of the sub-basins (see Supplementary Figures 6 and 7).

Decreased WP in the tropical western Atlantic and the Indian Oceans in Figure 3 in the main paper are associated to an El Niño weakening of the Walker circulation that reduces wind speeds in the equatorial Atlantic as the east and southeast trade winds decrease. El Niño is also followed by a weakened East Asian monsoon [13]. There is also historical evidence of a robust teleconnection of El Niño with the northern tropical Atlantic [14]. Although the mechanism is not yet fully understood, El Niño conditions are generally associated to cooler-than-average and more stable conditions in the northern tropical Atlantic, with less intense and frequent storms and extreme waves. This is consistent with the absence of patterns found in WP in Figures 3 and 4.

El Niño enforces convective motions and causes strengthened instability in the equatorial eastern Pacific, resulting in more intense and frequent storms and extreme winds [15–18]. This explains the significant positive correlation in Figures 3 and 4 between the tropical SST in the basin.

In the Indian ocean, patterns found in WP are also consistent with previous findings in Indian monsoon rainfall that has significant positive correlations with the Indian Ocean SST and moisture flux transport in the preceding winter and spring [19].

The dominant patterns in the wave climate are, in the Atlantic, the North Atlantic Oscillation [12] and the Artic Oscillation [3]; in the Southern Ocean, the Southern Annular Mode [20]; and in the Pacific the North American pattern, the West Pacific, El Niño Southern Oscillation (ENSO) and the Pacific Decadal Oscillation (PDO) [21–26]. WP decadal changes have been associated with

wave activity during different phases of these climate patterns, for example in the Pacific during the warm phase of the Pacific Decadal Oscillation [27,28].

The principal mode of variability of the extratropical atmospheric circulation in the Southern Hemisphere is the Southern Annular Mode (SAM). During its positive phase, the westerlies are enhanced in the Southern Ocean, with strong effects on wave climate across the whole Southern Ocean: wave heights increase and wave energy rotates clock-wisely as storm belts intensified in the region [20].

**Supplementary Code. Mann-Kendall test following the method in Wang and Swail (2001)**

Iterative method used to avoid autocorrelation in the Mann-Kendall test following the method in Wang and Swail (2001) [1]. The approach is implemented as described below [1]. For function *ktaub*, see [29].

```
function [taub tau h sig Z S sigma sen n senplot CIlower CIupper D Dall C3] =
WS2001_ktaub(t,Yt,alpha,wantplot)

% STEP 1)
c0 = autocorr(Yt); c0 = c0(2);
t1 = t(2:end);

if c0<0.05
[taub tau h sig Z S sigma sen n senplot CIlower CIupper D Dall C3] = ktaub([t(:) Yt(:)], alpha,
makeplot)
else
   Wt = (Yt(2:end) - c0.*Yt(1:end-1))./(1-c0);
   [fitresult, ~] = fit( t(2:end), Wt, 'poly1');
   [temp] = coeffvalues(fitresult);
   b0 = temp(1); %a0=temp(2);

   Dc= 1;
   Db= 1;

   while Dc>0.01 || Db>0.01

      % STEP 2
      Yt1 = Yt(2:end) - b0.*t1;
      c1 = autocorr(Yt1,1); c1 = c1(2);
      if c1<0.05,
         c = c0;
```

```
            b = b0;
            break
        else
            Wt = (Yt(2:end) - c1.*Yt(1:end-1))./(1-c1);
            [fitresult, ~] = fit( t1, Wt, 'poly1');
            [temp] = coeffvalues(fitresult);
            b1 = temp(1); % a1=temp(2);

            Dc = abs(c1-c0);
            Db = abs(b1-b0);

            disp(Dc)
            disp(Db)

            if Dc<=0.01 && Db<=0.01
                c = c1;
                b = b1;
            else
                c0 = c1;
                b0 = b1;
            end
        end
    end

    close all
    Wt = (Yt(2:end) - c.*Yt(1:end-1))./(1-c);
    [taub tau h sig Z S sigma sen n senplot CIlower CIupper D Dall C3] = ktaub([t1 Wt(:)], alpha,
wantplot)
end
end
```

## Supplementary References

1. Wang, X. L. & Swail, V. R. Changes of Extreme Wave Heights in Northern Hemisphere Oceans and Related Atmospheric Circulation Regimes. *J. Clim.* **14,** 2204–2221 (2001).
2. Sterl, A. & Caires, S. Climatology, variability and extrema of ocean waves: The web-based KNMI/ERA-40 wave atlas. *Int. J. Climatol.* **25,** 963–977 (2005).
3. Izaguirre, C. *et al.* Global extreme wave height variability based on satellite data. *Geophys. Res. Lett.* **38,** 1–6 (2011).
4. Méndez, F., Menendez, M., Luceno, a. & Losada, I. J. Modelling the seasonal-to-interannual variability of extreme sea levels. *Geophys. Res. Abstr.* **9,** 4–6 (2007).
5. Vermeer, M. & Rahmstorf, S. Global sea level linked to global temperature. *Pnas* **2009,** 1–6 (2009).
6. Mentaschi, L., Vousdoukas, M. I., Voukouvalas, E., Dosio, A. & Feyen, L. Global changes of extreme coastal wave energy fluxes triggered by intensified teleconnection patterns. *Geophys. Res. Lett.* **44,** 2416–2426 (2017).
7. Inatsu, M., Mukougawa, M. & Xie, S. Tropical and extratropical SST effects on the midlatitude storm track. *J. Meteorol. Soc. Japan* 1069–1076 (2002).
8. Bengtsson, L., Hodges, K. I. & Roeckner, E. Storm tracks and climate change. *J. Clim.* **19,** 3518–3543 (2006).
9. Wolter, K. The Southern Oscillation in Surface Circulation and Climate over the Tropical Atlantic, Eastern Pacific, and Indian Oceans as Captured by Cluster Analysis. *J. Clim. Appl. Meteorol.* **26,** 540–558 (1987).
10. Patricola, C. M., Saravanan, R. & Chang, P. A teleconnection between Atlantic sea surface temperature and eastern and central North Pacific tropical cyclones. *Geophys. Res. Lett.* **44,** 1167–1174 (2017).
11. Gastineau, G. & Frankignoul, C. Influence of the North Atlantic SST Variability on the Atmospheric Circulation during the Twentieth Century. *J. Clim.* **28,** 1396–1416 (2014).
12. Woolf, D. K. K., Challenor, P. ~G. P. G. & Cotton, P. D. Variability and predictability of the North Atlantic wave climate. *J. Geophys. Res.* **107(C10),** 3145 (2002).
13. Li, T., Zhang, Y., Chang, C.-P. & Wang, B. On the relationship between Indian Ocean sea surface temperature and Asian Summer Monsoon. *Geophys. Res. Lett.* **28,** 2843–2846 (2001).

14. Chang, P., Fang, Y., Saravanan, R., Ji, L. & Seidel, H. The cause of the fragile relationship between the Pacific El Nino and the Atlantic Nino. *Nature* **443,** 324–328 (2006).
15. Jin, F.-F., Boucharel, J. & Lin, I.-I. Eastern Pacific tropical cyclones intensified by El Nino delivery of subsurface ocean heat. *Nature* **516,** 82–85 (2014).
16. Rasmusson, E. M. & Carpenter, T. H. Variations in tropical sea surface temperature and surface winds fields associated with the Southern Oscillation/El Ni{ñ}o. *Mon. Weather Rev.* **110,** 354–384 (1982).
17. Timmerman, a *et al.* Increased El Niño frequency in a climate model forced by future greenhouse warming. *Nature* **398,** 694–697 (1999).
18. Philander, S. G. H. El Nino Southern Oscillation phenomena. *Nature* **302,** 295–301 (1983).
19. Li, T., Zhang, Y., Chang, C.-P. & Wang, B. On the relationship between Indian Ocean sea surface temperature and Asian Summer Monsoon. *Geophys. Res. Lett.* **28,** 2843–2846 (2001).
20. Hemer, M. A., Church, J. A. & Hunter, J. R. Variability and trends in the directional wave climate of the Southern Hemisphere wave climate of the Southern Hemisphere. *Int. J. Climatol.* **30,** 475–491 (2010).
21. Gulev, S. K. & Grigorieva, V. Last century changes in ocean wind wave height from global visual wave data. *Geophys. Res. Lett.* **31,** (2004).
22. Menendez, M. *et al.* Variability of extreme wave heights in the northeast Pacific Ocean based on buoy measurements. *Geophys. Res. Lett.* **35,** 1–6 (2008).
23. Bromirski, P. D., Cayan, D. R., Helly, J. & Wittmann, P. Wave power variability and trends across the North Pacific. *J. Geophys. Res. Ocean.* **118,** 6329–6348 (2013).
24. Reguero, B. G., Méndez, F. J. & Losada, I. J. Variability of multivariate wave climate in Latin America and the Caribbean. *Glob. Planet. Change* **100,** 70–84 (2013).
25. Harley, M. D., Turner, I. L., Short, A. D. & Ranasinghe, R. Interannual variability and controls of the Sydney wave climate. *Int. J. Climatol.* **30,** 1322–1335 (2010).
26. Shimura, T., Mori, N. & Mase, H. Ocean Waves and Teleconnection Patterns in the Northern Hemisphere. *J. Clim.* **26,** 8654–8670 (2013).
27. Reguero, B. G., Losada, I. J. & Méndez, F. J. A global wave power resource and its seasonal, interannual and long-term variability. *Appl. Energy* **148,** 366–380 (2015).

28. Bromirski, P. D., Cayan, D. R. & Flick, R. E. Wave spectral energy variability in the northeast Pacific. *J. Geophys. Res.* **110,** (2005).
29. Burkey, J. A non-parametric monotonic trend test computing Mann-Kendall Tau, Tau-b, and Sen's Slope written in Mathworks-MATLAB implemented using matrix rotations. (2006).
